# Incidence and effect of secondary cardiac amyloidosis on outcomes of patients with t(11;14) multiple myeloma

**DOI:** 10.3389/fcvm.2022.994384

**Published:** 2022-09-02

**Authors:** Jinghua Wang, Shuo Yang, Pengjun Liao, Lingji Zeng, Wei Ling, Li Wan, Jianyu Weng, Liye Zhong

**Affiliations:** ^1^Department of Hematology, Guangdong Provincial People's Hospital, Guangdong Academy of Medical Sciences, Guangzhou, China; ^2^Department of Hematology, Peking University Shenzhen Hospital, Shenzhen, China; ^3^Department of Endocrinology & Metabolism, Renmin Hospital of Wuhan University, Wuhan, China

**Keywords:** multiple myeloma, cardiac amyloidosis, heart failure, survival, risk factor

## Abstract

**Background:**

The t(11;14)(q13;32) is a common chromosome translocation in multiple myeloma (MM), but its prognostic value remains controversial. Immunoglobulin light chain amyloidosis is commonly secondary to multiple myeloma, which can rapidly cause heart failure and high mortality. We aimed to investigate the prevalence of secondary cardiac amyloidosis in MM patients with t(11;14) and to evaluate its impact on survival outcomes.

**Methods:**

We retrospectively identified 52 MM patients with t(11;14) in our center between October 2015 and April 2022. The associations between cardiac amyloidosis and clinical and biological parameters were statistically analyzed, and the impacts of concomitant of cardiac amyloidosis on survival and prognosis of MM patients with t(11;14) were also assessed.

**Results:**

Concomitant presence of cardiac amyloidosis was observed in 15 (28.8%) of all cases. Patients with cardiac amyloidosis had significantly higher NT-proBNP (*p* = 0.002) and higher hs-cTnT (*p* < 0.001), while the patients without cardiac amyloidosis had higher percentage of bone marrow plasma cells (*p* = 0.027), higher incidence of hemoglobin <80 g/L (*p* = 0.021) and bone destruction (*p* < 0.001). The median overall survival (OS) for all patients was 33.4 months after a median follow-up of 23.8 months. The amyloidosis group showed a significantly shorter OS than the non-amyloidosis group (15.3 vs. 41.8 months, *p* < 0.001). Besides, patients harboring NT-proBNP >1,800 pg/ml (*p* < 0.001) or hs-cTnT ≧40 pg/ml (*p* = 0.001) or light chain (LC) only isotype (*p* = 0.033) had a significantly shorter mean OS compared with patients with lower NT-proBNP or hs-cTnT or other M-protein isotype. Univariate analyses showed that NT-proBNP >1,800 pg/ml, hs-cTnT ≧40 pg/ml, LC only isotype, and concomitant presence of cardiac amyloidosis were independently associated with shorter OS, while NT-proBNP >1,800 pg/ml still retained the prognostic value for OS in multivariate analyses.

**Conclusion:**

The t(11;14) MM patients with coexisting cardiac amyloidosis may represent a distinct clinical entity that confers a poor outcome. These findings may have important clinical and biological implications.

## Introduction

Multiple myeloma (MM) is a malignant neoplasm characterized by clonal proliferation of malignant plasma cells in bone marrow (1). It accounts for about 10% of all hematologic malignancies and is the second most common hematologic malignancy after non-Hodgkin lymphoma (2). In MM, detection of cytogenetic abnormality is essential for predicting patient prognosis and disease management. The t(11;14)(q13;q32) is a frequently occurring chromosome translocation in MM and presents in 15–24% of newly diagnosed cases (3–6). This translocation cause IGH/CCND1 fusion and transcriptional activation of CCND1 oncogene by the IGH promoter/enhancer sequences, which contribute to multiple myeloma biology (6). The influence of t(11;14) on outcome remains controversial. Historically, the presence of t(11;14) was associated with relatively favorable outcomes, and patients with t(11;14) have been classified as standard risk MM up to now (3, 7–10). However, recent studies have challenged this paradigm with the conclusion that the prognosis of patients with t(11;14) is less favorable (11–13). These debatable results suggest that additional vital factors may affect the outcomes of t(11;14) MM, which should be managed accordingly.

Immunoglobulin light chain amyloidosis (AL) has long been recognized as a complication of MM. This complication results from the extracellular deposition of β-pleated sheet amyloid proteins which are consist of abnormal monoclonal immunoglobulin light chains (LC) secreted by abnormal plasma cells. These amyloid proteins are resistant to degradation and can infiltrate multiple organs, causing progressive organ dysfunction and death (14). Researches have suggested that up to 38% of MM patients may have subclinical AL, and 10–15% of patients may develop clinically overt AL during the course of the disease (15–19). Despite impressive therapeutic advances in the field of MM, MM-associated AL was also considered to be an independent high-risk prognostic factor for MM patients even in the absence of symptoms at diagnosis (18). The heart is the most commonly involved organ and cardiomyopathy appears in ~70% of AL patients (20). Moreover, asymptomatic patients with echocardiographic evidence of cardiac involvement usually develop cardiac symptoms later (21). Studies have shown that the survival time of patients with heart failure caused by cardiac amyloid deposition is much shorter than that of patients without heart failure (22). Therefore, the existence and extent of cardiac involvement is the key determinant of the prognosis of AL patients. However, there have been few studies regarding the concomitant presence of cardiac amyloidosis and t(11;14) MM, and it is unclear about the incidence rate of secondary cardiac amyloidosis in t(11;14) patients and its impact on the prognosis.

To address these issues, we examined the prevalence and clinical implications of secondary cardiac amyloidosis in a cohort of 52 MM patients with t(11;14), and assessed the impact of concomitant cardiac amyloidosis on patient survival outcomes. These findings may provide valuable information for the prognosis and optimal treatment of MM patients with t(11;14).

## Materials and methods

### Patients

This was a retrospective, cross-sectional, medical record review. A total of 52 consecutive MM patients with t(11;14) detected on fluorescence *in situ* hybridization (FISH) between October 2015 and April 2022 at Guangdong Provincial People's Hospital were included in this study. The diagnosis was made according to the 2014 International Myeloma Working Group criteria (23). The symptomatic MM and smoldering MM were both included in this study. The staging was made according to the International Staging System (ISS) and revised ISS (R-ISS), where the required laboratory parameters were available (24, 25). Their medical information regarding demographic characteristics, laboratory findings, treatment, response to induction, survival status, etc were collected in accordance with the Declaration of Helsinki. X-ray, computed tomography, magnetic resonance imaging (MRI), or positron emission tomography/computed tomography were used to determine bone destruction. This study was approved by the Ethics Committee of the Guangdong Provincial People's Hospital.

### Fluorescence *in situ* hybridization

All the patients had complete interphase FISH (iFISH) studies available. iFISH analysis was performed on CD138-purified plasma cells using probe sets for the detection of deletions, numerical aberrations and translocations. A total of 200 interphase nuclei were analyzed for each probe set, and the cut-offs recommended by the European Myeloma Network were used: for deletions and numerical aberrations, the cut-off level was set at 20%; for translocations in the IgH locus as well as other translocations, the cut-off level was set at 10% (10).

### Diagnosis of amyloidosis

Cardiac amyloidosis is diagnosed when the below criteria are met: a. tissue or organ biopsy proven amyloidosis and b. typical cardiac imaging features or/and abnormal cardiac biomarkers: abnormal age-adjusted NT-proBNP or abnormal hs-Troponin with all other causes for these changes excluded. The echocardiographic evidence indicates cardiac amyloidosis, usually manifested as ventricular wall thickening, poor diastolic filling, and abnormal longitudinal strain. The cardiac MRI evidence indicates cardiac amyloidosis, usually manifested as ventricular wall thickening, diffuse late gadolinium enhancement, and increased global extracelullar volume.

Amyloid deposits were established by histologic examination with Congo red staining in bone marrow, abdominal fat, or organ biopsy. If the stain was positive, and apple-green birefringence was observed under polarized microscopy, the patient was considered to have amyloid. Immunohistochemistry or immunofluorescence of the monoclonal LCs or proteomic analysis with mass spectrometry was performed in selected cases. The organ involvement for amyloidosis was assessed as follows: kidney (24-h urine protein >0.5 g/day, predominantly albumin), liver (total liver span >15 cm in the absence of heart failure or alkaline phosphatase >1.5 times institutional upper limit of normal), nerve (symmetric lower extremity sensorimotor peripheral neuropathy, gastric-emptying disorder, pseudo-obstruction, voiding dysfunction not related to direct organ infiltration), gastrointestinal tract (direct biopsy verification with symptoms), soft tissue (direct biopsy verification) (26).

### Response and outcome

Patients were treated with different induction therapy regimens, and some patients received autologous stem cell transplantation (ASCT). The induction regimens include bortezomib and dexamethasone, or bortezomib and dexamethasone plus cyclophosphamide, or bortezomib and dexamethasone plus thalidomide, or bortezomib and dexamethasone plus lenalidomide, etc. Response to therapy was assessed using the International Myeloma Working Group uniform response criteria (27). Overall survival (OS) was defined as the time from diagnosis of MM to the time of death or last follow-up. The cut-off date for follow-up was April 2022.

### Statistical analysis

Statistical analysis was conducted using SPSS software version 19. We summarized categorical variables as proportions and continuous variables as median (range). Categorical variables were compared using Chi-squared test or Fisher's exact, and continuous variables were compared using Student's *t*-test or Mann-Whitney *U*-test. Survival was analyzed using the Kaplan-Meier method and compared between groups using the log-rank test. Patients who were alive at the time of analysis were censored on the date of the last follow-up. Univariate and multivariate analyses of independent factors for OS were performed using the Cox proportional hazard model. A two-sided *p*-value < 0.05 was considered significant for all statistical tests.

## Results

### Patient characteristics

Table 1 shows the clinical characteristics and treatments of 52 patients with MM carrying t(11;14) included in our study. Briefly, the median age was 65 years and 63% were males. Most of the patients were LC only isotype, followed by IgG isotype, three were non-secretory, and one was smoldering MM. The LC of λ and κ was in 52% of patients and 40% of patients, respectively. The median bone marrow plasma cells were 32%. According to ISS and R-ISS stages, most had favorable features, such as low ISS and R-ISS stage (69% ISS I–II, 82% R-ISS I–II). About the cytogenetic abnormalities, del(17p) and 1q21 gain were detected in 5.8% and 28.8% of patients, respectively.

**Table 1:** Patient characteristics.

**Characteristics**	**Number of patients, *n* (%) or median (range)**
Age, years, median (range)	65 (39–83)
Male gender, *n* (%)	33 (63.5)
Hemoglobin, g/L, median (range)	96 (54–151)
Serum albumin, g/L, median (range)	32 (17–43)
β_2_-microglobulin, mmol/L, median (range)	6.78 (1.25–27.78)
LDH, U/L, median (range)	224 (15–593)
Calcium, mmol/L, median (range)	2.36 (82–3.20)
Ccr, ml/min/1.73 m^2^, median (range)	55 (5–139)
Abnormal FLC ratio (<0.26 or >1.65), *n* (%)	47 (90.4)
BMPCs, %, median (range)	32 (3–94)
**M-protein isotype**, ***n*** **(%)**
IgG	20 (38.5)
IgA	3 (5.8)
IgD	1 (1.9)
LC only	24 (46.2)
Others	4 (7.7)
**LC isotype**, ***n*** **(%)**
κ	21 (40.4)
λ	27 (51.9)
**ISS stage**, ***n*** **(%)**
I	12 (23.1)
II	24 (46.2)
III	16 (30.8)
**R-ISS stage**, ***n*** **(%)**
I	9 (17.3)
II	34 (65.4)
III	9 (17.3)
Presence of bone destruction, *n* (%)	24 (46.2)
**Cytogenetic abnormalities**, ***n*** **(%)**
del(17p)	3 (5.8)
1q21 gain	15 (28.8)
t(4;14)	1 (1.9)
Concomitant of cardiac amyloidosis, *n* (%)	15 (28.8)
**Treatment regimen**, ***n*** **(%)**
VD	9 (17.3)
VCD	19 (36.5)
VTD or VRD	9 (17.3)
Others	15 (28.8)
Underwent ASCT, *n* (%)	2 (3.8)

LDH, lactate dehydrogenase; Ccr, creatinine clearance rate; FLC, free light chain; LC, free light chain; BMPCs, bone marrow plasma cells; LC, light chain; ISS, International Staging System; R-ISS, revised International Staging System; VD, bortezomib, dexamethasone; VCD, bortezomib, cyclophosphamide, and dexamethasone; VTD, bortezomib, thalidomide, and dexamethasone; VRD, bortezomib, lenalidomide, and dexamethasone; ASCT, autologous stem cell transplantation.

### Prevalence of cardiac amyloidosis in t(11;14) MM

For the cohort as a whole, the concomitant presence of cardiac amyloidosis was observed in 15 (28.8%) of all t(11;14) cases. The diagnosis of cardiac amyloidosis was established by demonstrating amyloid in various target organs or tissues including heart (one), liver (one), soft tissue (eight), gastrointestinal (two), and bone marrow (three). The involvement of other organs outside the heart was as follows: soft tissue (nine), kidney (seven), gastrointestinal tract (two), peripheral nerves (two), and liver (one). Overall, involvement in 1, 2, and 3 or more organs occurred in 2 (13.3%), 6 (40.0%), and 7 (46.7%) patients, respectively. Among the patients with cardiac amyloidosis, cardiac MRI evidence of amyloidosis and echocardiographic evidence of amyloidosis were detected in 10 and 11 patients, separately. The median interventricular septal thickness was 13 mm, and the median ejection fraction was 55% on echocardiography. Additionally, abnormal electrocardiogram were detected in 11 patients, including low voltage in the limb, bundle branch block, atrial arrhythmia, pseudo-infarct Q-waves, etc.

### Correlation of cardiac amyloidosis with other clinicobiological characteristics

Statistical analysis was performed to evaluate possible associations between cardiac amyloidosis and the clinical and biological parameters. Patients were separated into two groups based on the presence or absence of cardiac amyloidosis, and the results are summarized in Table 2. Compared with the patients without cardiac amyloidosis, patients with cardiac amyloidosis had significantly higher NT-proBNP (*p* = 0.002) and higher hs-cTnT (*p* < 0.001). Hemoglobin <80 g/L (*p* = 0.021) and bone destruction (*p* < 0.001) were significantly more common in the patients without cardiac amyloidosis, and higher percentage of bone marrow plasma cells (*p* = 0.027) was also observed in this group. The incidence of IgG isotype was higher in the amyloidosis negative group (*p* = 0.027), while the incidence of LC only isotype was higher in the amyloidosis positive group (*p* = 0.002). On the other hand, the patients with amyloidosis seemed to have preferential λ LC isotype, but there was no significant difference between the two groups. There were trends that the patients with cardiac amyloidosis had lower albumin level, higher lactate dehydrogenase and alkaline phosphatase levels, but the results were not statistically significant. The other characteristics including age, gender, β2-microglobulin, hypercalcemia, renal dysfunction, difference between involved and uninvolved free light chain (dFLC), and stages were similar between the two groups. In addition to high-risk IgH translocations, del(17p) and 1q21 gain are another high-risk cytogenetic abnormalities. However, there was no significant correlation between cardiac amyloidosis and the presence of del(17p) or 1q21 gain.

**Table 2:** Baseline characteristics according to the presence or absence of cardiac amyloidosis.

**Characteristics**	**Amyloidosis positive (*n* = 15)**	**Amyloidosis negative (*n* = 37)**	***p*-Value**
Age, years, median (range)	64.7 (46–82)	65.2 (39.0–83.0)	0.864
Gender-male, *n* (%)	12 (80.0)	21 (56.8)	0.203
Hemoglobin <80 g/L, *n* (%)	1 (6.7)	15 (40.5)	0.021^*^
Serum albumin <25 g/L, *n* (%)	4 (26.7)	5 (13.5)	0.419
β_2_-microglobulin >5.5 mmol/L, *n* (%)	4 (26.7)	12 (32.4)	0.752
LDH >250 U/L, *n* (%)	6 (40.0)	9 (24.3)	0.318
Calcium >2.75 mmol/L, *n* (%)	0 (0)	4 (10.8)	0.311
Ccr <40 ml/min/1.73 m^2^, *n* (%)	6 (40.0)	13 (35.1)	0.760
NT-proBNP >332 pg/ml, *n* (%)	15 (100.0)	19 (51.4)	0.002^*^
hs-cTnT ≧40 pg/ml, *n* (%)	12 (80.0)	5 (13.5)	<0.001^*^
ALP >187.5 U/L, *n* (%)	2 (13.3)	1 (2.7)	0.196
BMPCs, %, median (range)	20.9 (4.0–80.0)	36.6 (3.0–94.0)	0.027^*^
dFLC ≥180 mg/L, *n* (%)	10 (66.7)	22 (59.5)	1.000
**M-protein isotype**, ***n*** **(%)**
IgG	2 (13.3)	18 (48.6)	0.027^*^
LC only	12 (80.0)	12 (32.4)	0.002^*^
**LC isotype**, ***n*** **(%)**
κ	4 (26.7)	17 (45.9)	0.230
λ	11 (73.3)	16 (43.2)	0.068
**ISS stage**, ***n*** **(%)**
I	4 (26.7)	8 (21.6)	0.926
II	7 (46.7)	17 (45.9)	
III	4 (26.7)	12 (32.4)	
**R-ISS stage**, ***n*** **(%)**
I	2 (13.3)	7 (18.9)	0.823
II	11 (73.3)	23 (62.2)	
III	2 (13.3)	7 (18.9)	
Presence of bone destruction, *n* (%)	2 (13.3)	26 (70.3)	<0.001^*^
**Cytogenetic abnormalities**, ***n*** **(%)**
del(17p)	0 (0)	3 (8.1)	0.546
1q21 gain	3 (20.0)	12 (32.4)	0.503

LDH, lactate dehydrogenase; Ccr, creatinine clearance rate; NT-proBNP, N-terminal pro-B-type natriuretic peptide; hs-cTnT, high-sensitivity cardiac Troponin T; ALP, alkaline phosphatase; BMPCs, bone marrow plasma cells; dFLC, difference between involved and uninvolved free light chain; LC, light chain; ISS, international staging system; R-ISS, revised international staging system.

* p < 0.05.

### Treatment and response

Among all patients, most patients were treated with bortezomib-based regimens (75.0%), and 25.0% were treated with immunomodulator-based regimens. Only two patients (3.8%) received ASCT. There were no data available for three patients. A total of 17 out of 52 patients with available data achieved ≥ very good partial response.

### Overall survival

**Figure 1:**
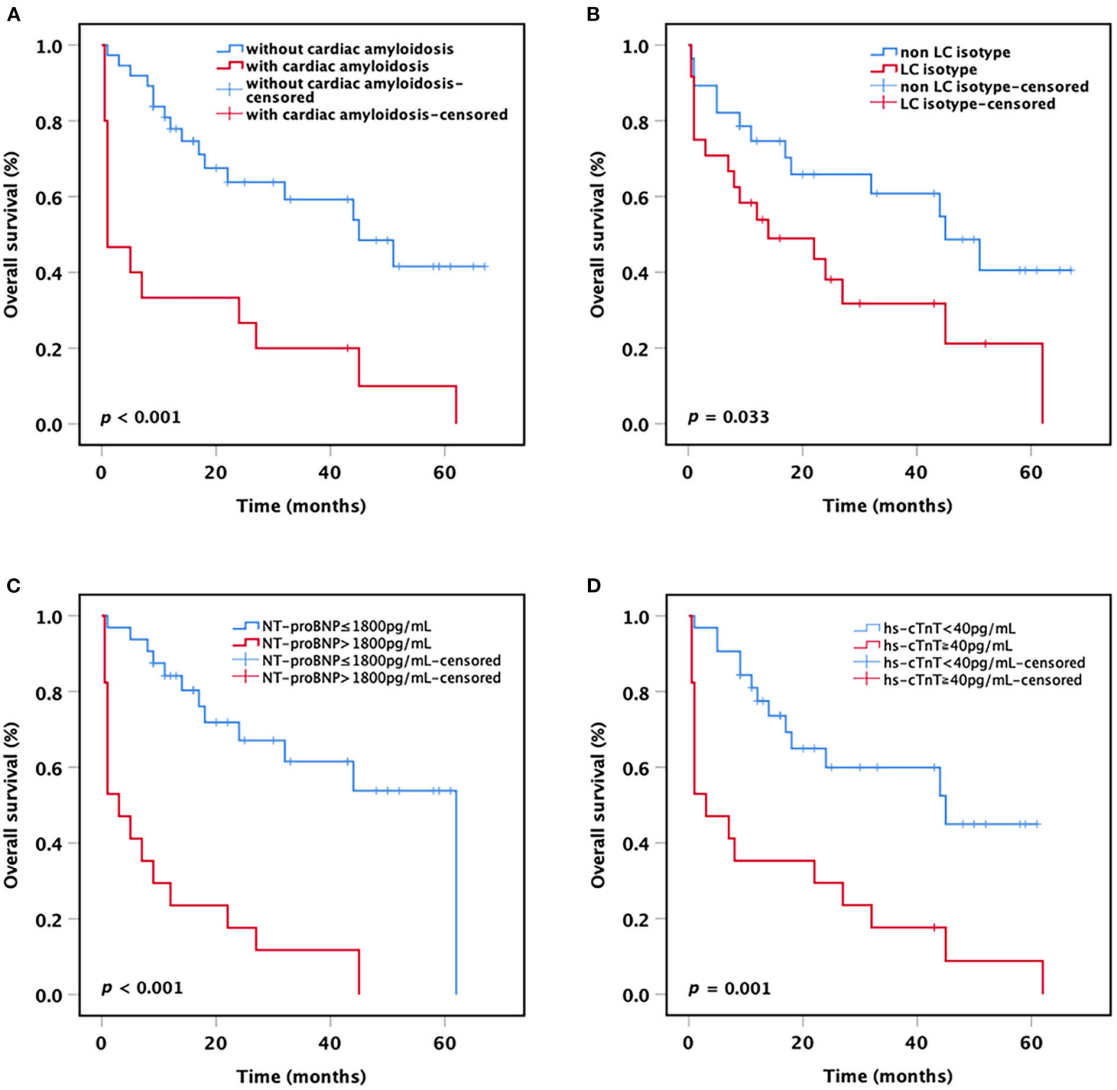
Kaplan-Meier curves. OS of t(11;14) MM patients **(A)** with or without cardiac amyloidosis, **(B)** with or without LC only, **(C)** with or without NT-proBNP >1,800 pg/ml, and **(D)** with or without hs-cTnT ≧40 pg/ml. OS, overall survival; LC, light chain; NT-proBNP, N-terminal pro-B-type natriuretic peptide; hs-cTnT, high-sensitivity cardiac Troponin T.

After a median follow-up of 23.8 months (range 0.5–67 months), the median OS for all patients was 33.4 months (95% CI 25.8–41.1 months). Among the 15 patients with concomitant presence of cardiac amyloidosis, 14 (93.3%) patients died during the follow-up period, and 8 (57.1%) patients died within 1 month after diagnosis. All these patients died of complications of amyloidosis. When patients were stratified based on the presence or absence of cardiac amyloidosis, the result showed that patients with cardiac amyloidosis had a significantly shorter mean OS compared with patients without cardiac amyloidosis (15.3 vs. 41.8 months, *p* < 0.001; Figure 1A). Besides, we found that the mean OS of patients with LC only isotype was shorter than that of patients with other M-protein isotypes (24.6 vs. 41.0 months, *p* = 0.033; Figure 1B).

As the current staging systems for systemic AL amyloidosis are based on the levels of circulating markers of cardiac and dFLC, we further analyzed the impact of cardiac biomarkers levels on the survival of all MM patients with t(11;14). The results showed that patients harboring NT-proBNP >1,800 pg/ml or hs-cTnT ≧40 pg/ml had a significantly shorter mean OS compared with patients with lower NT-proBNP or hs-cTnT (NT-proBNP >1,800 pg/ml vs. NT-proBNP ≤ 1,800 pg/ml: 12.3 vs. 41.3 months, *p* < 0.001; hs-cTnT ≧40 pg/ml vs. hs-cTnT <40 pg/ml: 15.6 vs. 39.1 months, *p* = 0.001; Figures 1C,D). Nevertheless, no significant difference was observed in OS between the patients with dFLC >180 mg/L and the patients with dFLC ≤ 180 mg/L (32.2 vs. 37.1 months, *p* = 0.463), probably due to the small sample size.

### Cox regression analysis

Univariate and multivariate analyses of potential prognostic factors for OS are summarized in Table 3. In the univariate analyses, NT-proBNP >1,800 pg/ml (HR = 5.510, *p* < 0.001), hs-cTnT ≧40 pg/ml (HR = 3.385, *p* = 0.001), LC only isotype (HR = 2.152, *p* = 0.040), and concomitant of cardiac amyloidosis (HR = 3.591, *p* = 0.001) were associated with significantly shorter OS (Table 2). In the multivariate analysis, all the parameters with a *p* < 0.05 in the univariate analysis for OS were included in the Cox model. The result showed that only NT-proBNP >1,800 pg/ml was significantly independent factors for shorter OS (HR = 3.965, *p* = 0.003).

**Table 3:** Univariate and multivariate survival analysis.

**Variable**	**Univariate**		**Multivariate**	
	**HR (95% CI)**	***p*-Value**	**HR (95% CI)**	***p*-Value**
Serum albumin <35 g/L	0.814 (0.379–1.747)	0.597	–	–
β_2_-microglobulin >5.5 mmol/L	1.576 (0.758–3.275)	0.223	–	–
LDH >250 U/L	1.186 (0.554–2.540)	0.660	–	–
NT-proBNP >1,800 pg/ml	5.510 (2.551–11.899)	<0.001^*^	3.965 (1.614–9.740)	0.003^*^
hs-cTnT ≧40 pg/ml	3.385 (1.598–7.170)	0.001^*^	1.506 (0.632–3.589)	0.355
dFLC ≥180 mg/L	1.380 (0.574–3.316)	0.472	–	–
LC only isotype	2.152 (1.035–4.474)	0.040^*^	0.761 (0.282–2.056)	0.590
ISS III (vs. ISS I/II)	1.576 (0.758–3.275)	0.223	–	–
R-ISS III (vs. R-ISS I/II)	1.019 (0.415–2.502)	0.968	–	–
With 1q21 gain	0.649 (0.276–1.530)	0.323	–	–
Concomitant of cardiac amyloidosis	3.591 (1.735–7.433)	0.001^*^	1.844 (0.658–5.171)	0.245

LDH, lactate dehydrogenase; NT-proBNP, N-terminal pro-B-type natriuretic peptide; hs-cTnT, high-sensitivity cardiac Troponin T; dFLC, difference between the involved and uninvolved free light chain; LC, light chain; ISS, international staging system; R-ISS, revised international staging system; HR, hazard ratio; CI, confidence interval.

* p < 0.05.

## Discussion

Multiple myeloma is a heterogeneous disease both clinically and genetically. The t(11;14) is a frequently occurring chromosome translocation in MM, while the prognostic value of t(11;14) in MM remains controversial in the era of novel agents. This discrepancy suggests that it is essential to identify other clinical or biological factors affecting the outcomes of t(11;14) MM. In this study, a high incidence of secondary cardiac amyloidosis was observed in t(11;14) MM, and the concomitant presence of cardiac amyloidosis was demonstrated to adversely affect the prognosis of patients with t(11;14).

Both MM and AL are malignant plasma cell proliferative diseases with unique characteristics. Previous studies have shown that the t(11;14) is detected in 15–24% of MM, while in 40–60% of AL that is higher than MM (3–6, 28), then it atrracted our attention to conduct this study to investigate whether the MM patients with t(11;14) are more prone to AL amyloidosis. It is known that the heart is the most common involved organ in AL amyloidosis, and the presence of cardiac involvement is the most significant prognostic factor for AL (14). Therefore, we concentrated on the cardiac amyloidosis in the present study.

In this single-center retrospective study, we investigated 52 patients with t(11;14) MM, and found that the prevalence of secondary cardiac amyloidosis was 28.8%. It should be noted that all the patients with cardiac amyloidosis in this study were clinically overt, and the prevalence of subclinical cardiac amyloidosis was perhaps higher. According to previous reports, clinically overt AL involving various organs accounted for up to 15% of all MM patients (15–19). This indicates to us that the incidence of amyloidosis is increased in the t(11;14) group, or the patients with t(11;14) MM are more prone to amyloidosis. The result also indicates that clinicians should have a high index of suspicion for cardiac amyloidosis in t(11;14) patients, especially those presenting with abnormal clinical or laboratory cardiac indicators.

Cardiac amyloidosis can present as restrictive cardiomyopathy, arrhythmia and heart failure comprise of systolic and diastolic heart failure (29). When a patient exhibits signs of palpitations, dyspnea on exertion, orthopnea, or syncope, and the laboratory finding shows abnormal increase of NT-pro BNP or hs-Troponin, or the cardiac imaging indicates amyloidosis, the presence of cardiac amyloidosis should be suspected. The next is to search for evidence of amyloidosis through biopsy specimens, and the biopsy site can include subcutaneous tissue, labial salivary gland, rectal mucosal, kidney and so on, which are common deposition sites of amyloid protein. In the present study, the most frequently selected site is soft tissue (53.3%) including subcutaneous tissue and labial salivary gland, which are minimally invasive. Endocardial biopsy is the gold standard method for the diagnosis of cardiac amyloidosis, but it is invasive and requires much experience (30). There was only one patient who received an endocardial biopsy in this study. Besides, the kidney was the most frequently affected organ besides the heart, which was consistent with previous reports (14).

When the patients were separated into two groups based on the presence or absence of cardiac amyloidosis, the results showed that the patients with cardiac amyloidosis had significantly higher NT-proBNP (*p* = 0.002) and higher hs-cTnT (*p* < 0.001) than the patients without amyloidosis, indicating increased incidence of heart failure in cardiac amyloidosis. Additionally, we found the patients with cardiac amyloidosis showed relatively lower tumor burden compared with the patients without amyloidosis, which was reflected in the lower percentage of bone marrow plasma cells, lower incidence of severe anemia, and bone destruction. This characterastics is similar to systemic AL manifests as lower tumor burden but systemic organ damage. According to previous reports, the patients with t(11;14) had a significantly higher proportion of LC only isotype (35%) in comparison to other cytogenetic abnormalities (4, 31). In our study, LC only isotype was also demonstrated to be the most common M-protein isotype for the whole t(11;14) cohort (46.2%), meanwhile, we found that the LC only isotype showed closely associated with cardiac amyloidosis (cardiac amyloidosis vs. no cardiac amyloidosis: 80.0% vs. 32.4%, *p* = 0.002). These results suggest that MM patients with amyloidosis may produce more pathogenic free light chain (FLC) causing symptoms of systemic AL, even before the development of symptomatic MM. Futhermore, it is reported that the λ LC isotype accounts for the majority of systemic AL (32), and λ predominance was also observed in patients with amyloidosis in our study, but not statistically significant, probably due to the small sample size.

We know that the most important prognostic factor in patients with systemic AL is whether there is cardiac involvement, and sudden cardiac death is an important cause of high early mortality in AL patients (20). Nevertheless, there was limited data about the impact of cardiac amyloidosis on the survival of MM patients. In this study with t(11;14) MM, we found that the patients with cardiac amyloidosis had a significantly shorter mean OS compared with patients without cardiac amyloidosis (15.3 vs. 41.8 months, *p* < 0.001). We also found that there were 8 (57.1%) patients who died of complications of amyloidosis within 1 month after diagnosis. Moreover, the concomitant presence of cardiac amyloidosis was demonstrated to be an independent factor for shorter OS in univariate analysis (HR = 3.591, *p* = 0.001). These results matched our priors that the t(11;14) myeloma represents a heterogeneous disease rather than a unique clinical and biological entity. Not all patients with t(11;14) belong to “standard risk disease,” which need comprehensive evaluation. Once the patients are complicated with cardiac amyloidosis, the prognosis will be very poor, and the presence of secondary cardiac amyloidosis may be a good risk-stratification factor needed to be validated further.

Staging of AL amyloidosis is based on NT-proBNP >1,800 pg/ml, hs-cTnT ≧40 pg/ml, and dFLC ≥180 mg/L (14), we also evaluated the value of these three indicators in the prognosis of t(11;14) MM. The results showed that NT-proBNP >1,800 pg/ml (HR = 5.510, *p* < 0.001) and hs-cTnT ≧40 pg/ml (HR = 3.385, *p* = 0.001) were independent factors for shorter OS in univariate analyses, and NT-proBNP >1,800 pg/ml retained the prognostic value for OS in multivariate analyses (HR = 3.965, *p* = 0.003). Our study did not find a significant correlation between the level of dFLC with OS in t(11;14) MM.

Additionally, we found that the patients with LC only isotype had shorter mean OS compared with patients harbor other M-protein isotype (*p* = 0.033), and LC only isotype was an independent factor for shorter OS in univariate analysis (HR = 3.591, *p* = 0.040). These results indicate that the LC only isotype may represent a poorer prognosis of the disease. Of note, ISS, R-ISS, and other high-risk cytogenetic abnormalities like 1q21 lost their prognostic values in our study, indicating that the t(11;14) MM requires a unique staging system that takes into account cardiac function indicators.

The outcomes for patients with MM have improved significantly in the era of novel agents (33), most of the patients also received novel agent therapies including bortezomib and immunomodulators in our study, nevertheless, the patients with secondary cardiac amyloidosis were unlikely to benefit from therapy and had a high early death. Therefore, we need to explore the optimal management for this complication in order to improve the prognosis. Rapid and profound elimination of pathogenic FLC is essential to ameliorate cardiac function, so it will be valuable to assess the potential benefits of various induction protocols and ASCT in this disease. BCL2 inhibition venetoclax has demonstrated robust single-agent activity in t(11;14) MM that harbor high expression of antiapoptotic protein BCL-2, which can be evaluated in t(11;14) with cardiac amyloidosis as well in the future (31). Moreover, immunotherapies targeting the amyloid deposits are undergoing clinical trials, which are expected to reverse organ dysfunction (20).

To the best of our knowledge, this is the first study to find an association between the concomitant of cardiac amyloidosis in t(11;14) MM and shorter OS. Limitations of this study include the small sample size of patients, large data missing, lack of patients treated with other novel agents such as daratumumab, and lack of analysis with other coexisting high-risk factors. Additional studies with larger cohorts of patients are warranted to evaluate the clinical entity of secondary cardiac amyloidosis in MM patients with t(11;14).

## Conclusion

In summary, we observed a high prevalence of secondary cardiac amyloidosis in MM patients with t(11;14), and the presence of cardiac amyloidosis was a key determinant of patient survival that in need of optimal management.

## Data availability statement

The original contributions presented in the study are included in the article/supplementary material, further inquiries can be directed to the corresponding author/s.

## Ethics statement

The studies involving human participants were reviewed and approved by Ethics Committee of the Guangdong Provincial People's Hospital. The patients/participants provided their written informed consent to participate in this study. Written informed consent was obtained from the individual(s) for the publication of any potentially identifiable images or data included in this article.

## Author contributions

JWa and SY acquired the data, performed the analysis, and wrote the manuscript. PL participated in data analysis. LZe, LW, and WL were responsible for data curation. LZh and JWe were involved in study design, supervision, and acquiring funding. All authors contributed to the study conception, design, read, and approved the final manuscript.

## Funding

This work was supported by the National Natural Science Foundation of China (No. 82100238), the Science and Technology Program of Guangzhou (No. 202201011046), the High-level Hospital Construction Project (No. DFJH201923), and the Medical Scientific Research Foundation of Guangdong Province (No. A2019063).

## Conflict of interest

The authors declare that the research was conducted in the absence of any commercial or financial relationships that could be construed as a potential conflict of interest.

## Publisher's note

All claims expressed in this article are solely those of the authors and do not necessarily represent those of their affiliated organizations, or those of the publisher, the editors and the reviewers. Any product that may be evaluated in this article, or claim that may be made by its manufacturer, is not guaranteed or endorsed by the publisher.
